# Prognostic significance of the pre-chemotherapy lymphocyte-to-monocyte ratio in patients with previously untreated metastatic colorectal cancer receiving FOLFOX chemotherapy

**DOI:** 10.1186/s40880-015-0063-1

**Published:** 2016-01-06

**Authors:** Gui-Nan Lin, Pan-Pan Liu, Dong-Ying Liu, Jie-Wen Peng, Jian-Jun Xiao, Zhong-Jun Xia

**Affiliations:** Department of Medical Oncology, Zhongshan City People’s Hospital/Zhongshan Hospital of Sun Yat-sen University, Zhongshan, Guangdong 528400 P.R. China; Department of Medical Oncology, Sun Yat-sen University Cancer Center; State Key Laboratory of Oncology in South China, Collaborative Innovation Center for Cancer Medicine, Guangzhou, Guangdong 510060 P.R. China; Department of Clinical Oncology, Jiangmen City Central Hospital/Jiangmen Hospital of Sun Yat-sen University, Jiangmen, Guangdong 529071 P.R. China; Department of Hematologic Oncology, Sun Yat-sen University Cancer Center; State Key Laboratory of Oncology in South China, Collaborative Innovation Center for Cancer Medicine, Guangzhou, Guangdong 510060 P.R. China

**Keywords:** Metastatic colorectal cancer, Inflammation, Lymphocyte, Monocyte, Prognosis

## Abstract

**Background:**

As a surrogate marker of systemic inflammation, the lymphocyte-to-monocyte ratio (LMR) is an independent prognostic factor for various malignancies. This study investigated the prognostic significance of the pre-chemotherapy LMR in patients with previously untreated metastatic colorectal cancer (mCRC) receiving chemotherapy.

**Methods:**

The present study included newly diagnosed mCRC patients treated between January 2005 and December 2013 with FOLFOX chemotherapy, specifically oxaliplatin 180 mg/m^2^ on day 1, with leucovorin 400 mg/m^2^ administered as a 2-hour infusion before the administration of 5-fluorouracil 400 mg/m^2^ as an intravenous bolus injection, and 5-fluorouracil 2400 mg/m^2^ as a 46-h infusion immediately after 5-fluorouracil bolus injection. The LMR was calculated as the absolute count of lymphocytes divided by the absolute count of monocytes. COX proportional hazards analysis was performed to evaluate the association of LMR with survival outcomes.

**Results:**

A total of 488 patients were included. Patients with high pre-chemotherapy LMR experienced significant improvements in progression-free survival (PFS, 9.2 vs. 7.6 months, *P* < 0.001) and overall survival (OS, 19.4 vs. 16.6 months, *P* < 0.001) compared with patients with low pre-chemotherapy LMR. Subsequent COX multivariate analysis showed that high pre-chemotherapy LMR (≥3.11) was an independent favorable prognostic factor for PFS and OS. Additionally, patients whose LMR remained high (high–high subgroup), increased (low–high subgroup), or decreased (high–low subgroup) following chemotherapy showed better results in terms of PFS and OS than patients whose LMR remained low (low–low subgroup) after chemotherapy.

**Conclusions:**

For patients with previously untreated mCRC receiving FOLFOX chemotherapy, an elevated pre-chemotherapy LMR is an independent favorable prognostic factor for PFS and OS, and changes in the LMR before and after chemotherapy seem to predict the benefit of chemotherapy.

## Background

Colorectal cancer (CRC) is the third most frequently diagnosed cancer among men and women in the most developed areas in China, and its incidence is increasing significantly because of the aging population [1, 2]. Metastatic disease is found in approximately half of all patients [3]. Combined treatment with oxaliplatin (OHP) and 5-fluorouracil (5-FU) that is biochemically activated by leucovorin (LV), also known as FOLFOX regimen, remains the first-line treatment of metastatic colorectal cancer (mCRC) patients, with an objective response rate of 31% and a median survival of approximately 14 months [4, 5]. Due to unsatisfactory results obtained by palliative chemotherapy, identifying prognostic and predictive biomarkers that can be used to improve disease management through patient classification remains the subject of intense investigation in mCRC.

Inflammation is a key component of the tumor microenvironment [6]. Accumulating evidence has shed light on the molecular pathways that link inflammation and cancer, suggesting that inflammation in the tumor microenvironment leads to neoplastic cell proliferation, angiogenesis, metastasis, collapse of antitumor immunity, and unresponsiveness to antineoplastic therapy [7–9]. Therefore, cancer-related inflammation represents a potential therapeutic target for treating cancer [10]. Lymphocytes can turn into tumor-infiltrating lymphocytes (TILs) by migrating into the tumor microenvironment. Stage III CRC patients with a high level of TILs experience better results in terms of 5-year overall survival (OS) and disease-free survival (DFS) than patients with a low level of TILs [11]. Monocytes can differentiate into macrophages in the tumor microenvironment. In turn, macrophages promote tumor cell migration, invasion, and metastasis [12]. As a surrogate marker of systemic inflammation, the lymphocyte-to-monocyte ratio (LMR) serves as an independent prognostic factor for a large variety of malignancies [13–16]. A study including 372 patients with stages II and III colon cancer revealed that patients with preoperative LMR > 2.83 obtained significant improvements in terms of time-to-recurrence and OS compared with patients with LMR ≤ 2.83, and the benefit of adjuvant 5-FU-based chemotherapy was limited to patients with preoperative LMR > 2.83 [17].

To the best of our knowledge, the prognostic significance of circulating LMR in patients with previously untreated mCRC receiving FOLFOX chemotherapy is not well defined. Therefore, this study investigated the prognostic significance of pre-chemotherapy LMR in patients with newly diagnosed mCRC receiving FOLFOX chemotherapy.

## Patients and methods

### Ethical statement

This study was approved by the ethics committee of the Zhongshan Hospital of Sun Yat-sen University. Written informed consent was obtained from all patients prior to treatment.

### Patient selection

Between January 2005 and December 2013, patients treated at the Zhongshan Hospital of Sun Yat-sen University were retrospectively identified to determine their eligibility for the study. The eligibility criteria were as follows: age ≥18 and ≤75 years, pathologically proven and chemotherapy-naïve mCRC, Eastern Cooperative Oncology Group (ECOG) performance status of 0–2, first-line chemotherapy with FOLFOX (OHP 180 mg/m^2^ on day 1 with LV 400 mg/m^2^ administered as a 2-h infusion before 5-FU 400 mg/m^2^ administered as an intravenous bolus injection, and 5-FU 2400 mg/m^2^ as a 46-h infusion immediately after 5-FU bolus injection), chemotherapy duration ≥6 and ≤12 cycles, and complete clinical and follow-up data. Patients were excluded if they had any active infections prior to the first chemotherapy cycle.

### Measurement of LMR

Peripheral blood was obtained within 3 days before the initiation of the first cycle of chemotherapy and between 3 and 4 weeks after the completion of the last cycle. A complete blood count with differential quantification was generated using the automated hematology analyzer Sysmex XE-2100 (Sysmex, Kobe, Japan). The LMR was calculated by dividing the absolute lymphocyte count (ALC) by the absolute monocyte count (AMC).

### Statistical analysis

Progression-free survival (PFS) was calculated from the date of chemotherapy initiation to the date of first progression, death from any cause, or the last follow-up. OS was calculated from the time of diagnosis to the date of death from any cause. The optimal cutoff levels were determined by receiver operating curve (ROC) analysis. A Chi-square test was used to compare baseline features between different groups. Survival curves were generated using the Kaplan–Meier method and compared using the log-rank test. Univariate and multivariate analyses to determine prognostic predictors were performed using COX proportional hazard regression models. These analyses were performed with SPSS software (version 16.0, SPSS Inc., Chicago, IL, USA). A two-tailed *P* < 0.05 was considered significant.

## Results

### Patient characteristics

A total of 488 patients were eligible for the study. The median age at diagnosis was 54 years (range 37–72 years). The ratio of males to females was approximately 1.2:1. An ECOG performance status of less than 2 was observed in 314 patients (64.3%). Primary colon cancer was diagnosed in 259 patients (53.1%). The tumor was well or moderately differentiated in 283 patients (58.0%). The median number of metastatic sites was 2 (range 1–5). The median chemotherapy cycles were 9 (range 6–12). The median pre-chemotherapy lymphocyte and monocyte counts were 2.83 × 10^9^/L (range 0.31–5.64 × 10^9^/L) and 0.65 × 10^9^/L (range 0.16–4.19 × 10^9^/L), respectively. All patients were followed up until December 31, 2014. The median follow-up time was 23.5 months (range 4.3–32.8 months).

### Selection of cut-off values

Based on ROC curves for survival analysis, the optimal cut-off values were 2.70 × 10^9^/L for ALC, with an area under the curve (AUC) value of 0.634 [95% confidence interval (CI) 0.546–0.724, *P* = 0.034]; 0.55 × 10^9^/L for AMC, with an AUC value of 0.712 (95% CI 0.613–0.795, *P* = 0.008); and 3.11 for LMR, with an AUC value of 0.733 (95% CI 0.638–0.786, *P* = 0.004).

### Comparison of patient characteristics based on the cut-off values

Of the 488 patients, 216 were assigned to the low-LMR (LMR < 3.11) group, and 272 were assigned to the high-LMR (LMR ≥ 3.11) group. A comparison of the baseline patient characteristics between high- and low-LMR groups is presented in Table 1. Both groups were well balanced with respect to patient age, gender, and the site of primary tumor. However, significant differences between the low- and high-LMR groups were observed in terms of ECOG performance status, number of metastatic sites, differentiation, pre-chemotherapy ALC, and pre-chemotherapy AMC (all *P* < 0.05).

**Table 1:** Comparison of baseline clinical characteristics of patients with newly diagnosed metastatic colorectal cancer stratified by pre-treatment lymphocyte-to-monocyte ratio (LMR)

Characteristic	LMR < 3.11 [cases (%)]	LMR ≥ 3.11 [cases (%)]	*P* value
Total	216	272	
Gender			0.817
Male	119 (55.1)	147 (54.0)	
Female	97 (44.9)	125 (46.0)	
Age (years)			0.802
<60	139 (64.4)	178 (65.4)	
≥60	77 (35.6)	94 (34.6)	
ECOG performance status			0.013
<2	126 (58.3)	188 (69.1)	
≥2	90 (41.7)	84 (30.9)	
Site of primary tumor			0.907
Colon	114 (52.8)	145 (53.3)	
Rectum	102 (47.2)	127 (46.7)	
No. of metastatic sites			<0.001
≤2	157 (72.7)	118 (43.4)	
>2	59 (27.3)	154 (56.6)	
Tumor differentiation			<0.001
Well or moderate	163 (75.5)	120 (44.1)	
Poor	53 (24.5)	152 (55.9)	
Pre-chemotherapy ALC (×10^9^/L)			
<2.70	122 (56.5)	107 (39.3)	
≥2.70	94 (43.5)	165 (60.7)	
Pre-chemotherapy AMC (×10^9^/L)			<0.001
<0.55	57 (26.4)	128 (47.1)	
≥0.55	159 (73.6)	144 (52.9)	

*ALC* absolute lymphocyte count, *AMC* absolute monocyte count, *ECOG* Eastern Cooperative Oncology Group

### Survival outcomes

**Fig. 1:**
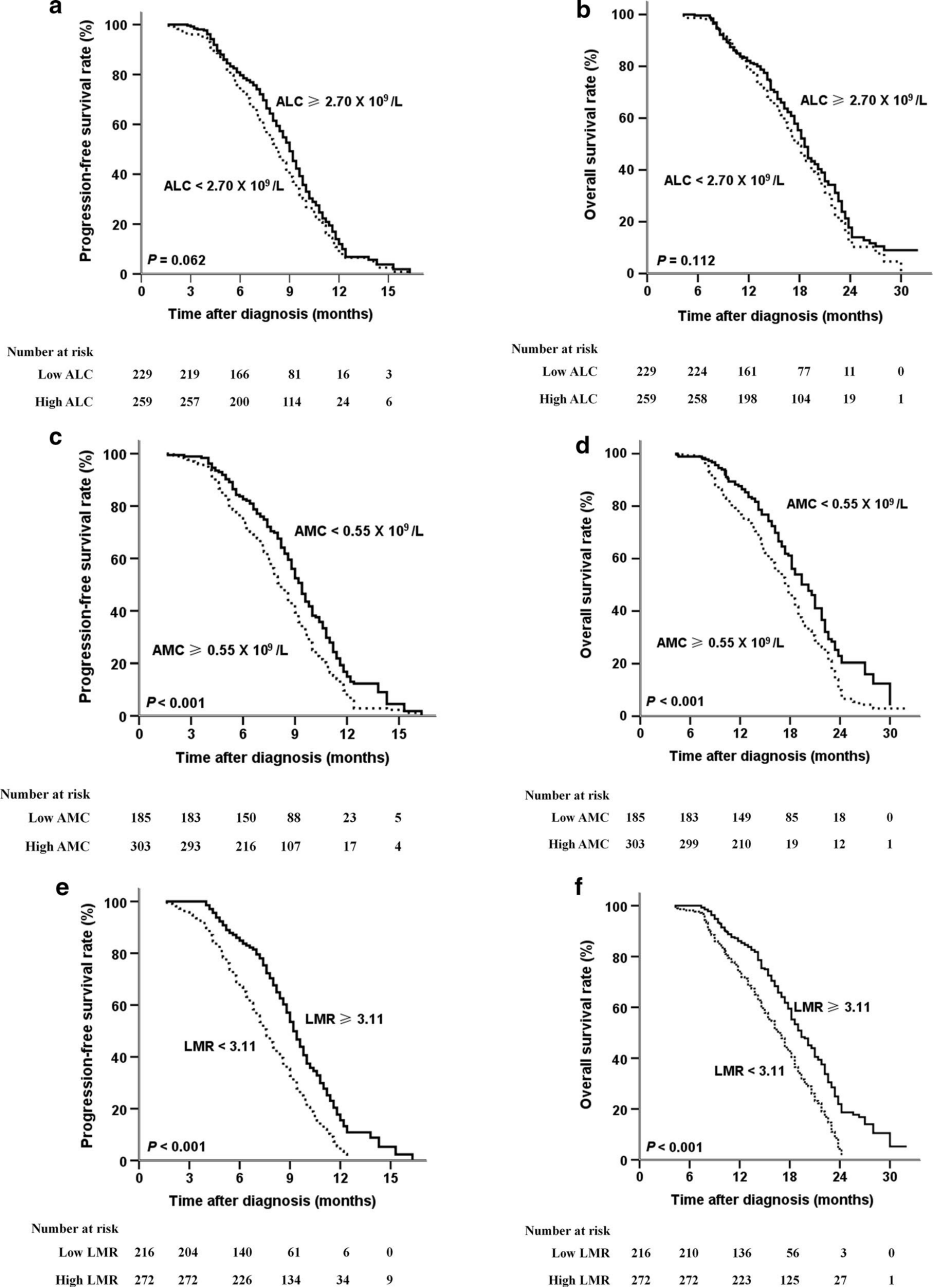
Kaplan–Meier estimates of progression-free and overall survival of patients with newly diagnosed metastatic colorectal cancer stratified by pre-treatment ALC (**a**, **b**), AMC (**c**, **d**), and LMR (**e**, **f**). *ALC* absolute lymphocyte count, *AMC* absolute monocyte count, *LMR* lymphocyte-to-monocyte ratio

The median PFS and OS for all patients were 8.8 months (95% CI 8.4–9.2 months) and 18.2 months (95% CI 17.5–18.9 months), respectively. Patients with a pre-chemotherapy ALC of 2.70 × 10^9^/L or greater had longer, though not significantly longer, PFS [9.0 months (95% CI 8.6–9.4 months) vs. 8.2 months (95% CI 7.6–8.8 months), *P* = 0.062; Fig. 1a] and OS [18.6 months (95% CI 17.9–19.3 months) vs. 17.8 months (95% CI 16.6–19.0 months), *P* = 0.112; Fig. 1b] compared with patients with an ALC of less than 2.70 × 10^9^/L. However, patients with a pre-chemotherapy AMC of less than 0.55 × 10^9^/L had significantly longer PFS [9.4 months (95% CI 8.9–9.9 months) vs. 8.2 months (95% CI 7.7–8.7 months), *P* < 0.001; Fig. 1c] and OS [20.2 months (95% CI 18.7–21.7 months) vs. 17.4 months (95% CI 16.3–18.5 months), *P* < 0.001; Fig. 1d] than patients with an AMC of 0.55 × 10^9^/L or greater. In addition, patients in the high-LMR group experienced significant improvements in PFS [9.2 months (95% CI 8.8–9.6 months) vs. 7.6 months (95% CI 7.0–8.2 months), *P* < 0.001; Fig. 1e] and OS [19.4 months (95% CI 18.0–20.8 months) vs. 16.6 months (95% CI 15.3–17.9 months), *P* < 0.001; Fig. 1f] compared with patients in the low-LMR group.

### Univariate and multivariate analysis of pre-chemotherapy LMR as a prognostic factor for PFS

Age, gender, ECOG performance status, site of primary tumor, number of metastatic sites, pre-chemotherapy ALC, AMC, and LMR were included in univariate and multivariate analyses. The univariate and multivariate analyses for PFS are shown in Table 2. Based on the univariate analysis, an ECOG performance status of less than 2, no more than 2 metastatic sites, well or moderate differentiation, low pre-chemotherapy AMC (<0.55 × 10^9^/L), or high pre-chemotherapy LMR (≥3.11) were significantly associated with longer PFS (all *P* < 0.01). In the subsequent multivariate analysis, independent unfavorable prognostic factors for PFS included a ECOG performance status of more than 2, more than 2 metastatic sites, poor differentiation, and high pre-chemotherapy AMC (all *P* < 0.05), whereas only high pre-chemotherapy LMR was an independent favorable prognostic factor for PFS (*P* = 0.005).

**Table 2:** Univariate and multivariate analyses of variables associated with progression-free survival of patients with newly diagnosed metastatic colorectal cancer

Characteristic	Univariate analysis		Multivariate analysis	
	HR (95% CI)	*P* value	HR (95% CI)	*P* value
Gender				0.931
Male	1 (reference)	0.870	1 (reference)	
Female	0.984 (0.815–1.189)		0.992 (0.819–1.200)	
Age (years)				0.837
<65	1 (reference)	0.931	1 (reference)	
≥65	0.991 (0.714–1.207)		0.837 (0.668–1.408)	
ECOG performance status				<0.001
<2	1 (reference)	0.009	1 (reference)	
≥2	1.300 (1.066–1.585)		1.515 (1.206–1.907)	
Site of primary tumor				0.560
Colon	1 (reference)	0.542	1 (reference)	
Rectum	1.060 (0.878–1.280)		1.058 (0.876–1.278)	
No. of metastatic sites				0.049
≤2	1 (reference)	0.001	1 (reference)	
>2	1.388 (1.148–1.680)		1.229 (1.001–1.509)	
Tumor differentiation				0.005
Well or moderate	1 (reference)	<0.001	1 (reference)	
Poor	1.617 (1.334–1.961)		1.354 (1.095–1.675)	
Pre-chemotherapy ALC (× 10^9^/L)				0.059
<2.70	1 (reference)	0.072	1 (reference)	
≥2.70	0.841 (0.697–1.015)		0.792 (0.621–1.009)	
Pre-chemotherapy AMC (× 10^9^/L)				0.001
<0.55	1 (reference)	0.001	1 (reference)	
≥0.55	1.409 (1.159–1.713)		1.513 (1.172–1.954)	
Pre-chemotherapy LMR				0.005
<3.11	1 (reference)	<0.001	1 (reference)	
≥3.11	0.552 (0.454–0.671)		0.710 (0.558–0.903)	

*HR* hazard ratio, *CI* confidential interval. Other abbreviations as in Table 1

### Univariate and multivariate analyses of pre-chemotherapy LMR as a prognostic factor for OS

The univariate and multivariate analyses for OS are summarized in Table 3. The univariate analysis revealed that an ECOG performance status of less than 2, no more than 2 metastatic sites, well or moderate differentiation, low pre-chemotherapy AMC (<0.55 × 10^9^/L), or high pre-chemotherapy LMR (≥3.11) were significantly linked to a favorable OS. Subsequently, the multivariate analysis demonstrated that an ECOG performance status of more than 2, poor differentiation, and high pre-chemotherapy AMC were independent prognostic factors for shorter OS (all *P* < 0.01), whereas high pre-chemotherapy LMR (≥3.11) was an independent prognostic factor for longer OS (*P* = 0.004).

**Table 3:** Univariate and multivariate analyses of variables associated with overall survival of patients with newly diagnosed metastatic colorectal cancer

Characteristic	Univariate analysis		Multivariate analysis	
	HR (95% CI)	*P* value	HR (95% CI)	*P* value
Gender		0.055		0.07
Male	1 (reference)		1 (reference)	
Female	0.804 (0.644–1.004)		0.812 (0.648-1.017)	
Age (years)		0.567		0.199
<65	1 (reference)		1 (reference)	
≥65	1.070 (0.849–1.347)		0.842 (0.647–1.095)	
ECOG performance status		0.005		<0.001
<2	1 (reference)		1 (reference)	
≥2	1.388 (1.103–1.747)		1.620 (1.244–2.111)	
Site of primary tumor		0.974		0.938
Colon	1 (reference)		1 (reference)	
Rectum	1.004 (0.806–1.250)		1.009 (0.809–1.257)	
No. of metastatic sites		0.002		0.065
≤2	1 (reference)		1 (reference)	
>2	1.417 (1.135–1.769)		1.252 (0.986–1.589)	
Tumor differentiation		<0.001		0.002
Well or moderate	1 (reference)		1 (reference)	
Poor	1.736 (1.385–2.176)		1.484 (1.159–1.901)	
Pre-chemotherapy ALC (×10^9^/L)		0.121		0.391
<2.70	1 (reference)		1 (reference)	
≥2.70	0.841 (0.676–1.047)		0.884 (0.668–1.171)	
Pre-chemotherapy AMC (×10^9^/L)		<0.001		<0.001
<0.55	1 (reference)		1 (reference)	
≥0.55	1.514 (1.204–1.903)		1.703 (1263–2.296)	
Pre-chemotherapy LMR		<0.001		0.004
<3.11	1 (reference)		1 (reference)	
≥3.11	0.568 (0.453–0.712)		0.662 (0.501–0.875)	

Abbreviations as in Tables 1 and 2

### Changes in LMR and benefits of chemotherapy

**Fig. 2:**
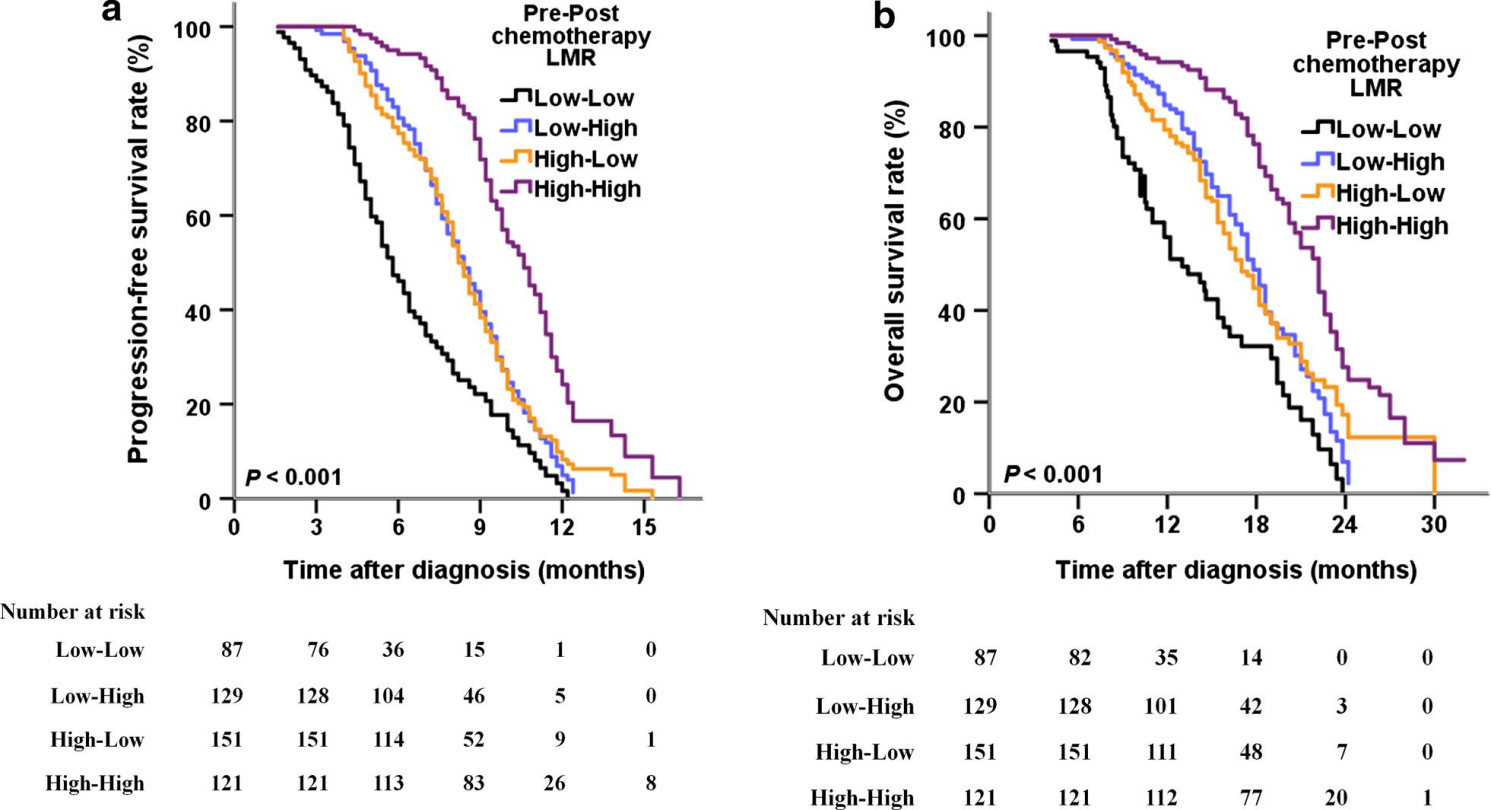
Kaplan–Meier estimates of progression-free and overall survival of patients with newly diagnosed metastatic colorectal cancer according to the changes in LMR before and after chemotherapy. **a** progression-free survival, **b** overall survival

The pretreatment low- and high-LMR groups were subdivided by the cut-off point of the post-chemotherapy LMR (3.11) as follows: low–low, low–high, high–low, and high–high. The changes in LMR and benefits of chemotherapy are listed in Table 4 and Fig. 2. Patients whose LMR remained high (high–high subgroup), increased (low–high subgroup), or decreased (high–low subgroup) after chemotherapy had significantly prolonged PFS and OS compared with patients whose LMR remained low (low–low subgroup).

**Table 4:** Changes in LMR and benefits of chemotherapy

Pre- and post-chemotherapy LMR	No. of patients	Progression-free survival (months)			Overall survival (months)		
		Median (95% CI)	HR (95% CI)	*P* value	Median (95% CI)	HR (95% CI)	*P* value*
Low–low	87	5.8 (5.1–6.5)	1		13.0 (10.3–15.7)	1	
Low–high	129	8.4 (7.7–9.1)	0.524 (0.391–0.702)	<0.001	17.8 (16.9–18.7)	0.522 (0.371–0.771)	<0.001
High–low	151	8.2 (7.8–8.6)	0.733 (0.636–0.846)	<0.001	17.0 (15.6–18.4)	0.837 (0.699–1.003)	0.053
High–high	121	10.6 (9.8–11.4)	0.631 (0.566–0.702)	<0.001	22.2 (21.0–23.4)	0.636 (0.562–0.719)	<0.001

Abbreviations as in Tables 1 and 2

*Tested by COX proportional hazards model in which the reference is the low–low group, and adjusted with the ECOG performance status, number of metastatic sites, and differentiation

## Discussion

In the present study, patients with high pre-chemotherapy LMR experienced significant improvements in PFS (9.2 vs. 7.6 months, *P* < 0.001) and OS (19.4 vs. 16.6 months, *P* < 0.001) compared with patients with low pre-chemotherapy LMR. Subsequent COX multivariate analysis showed that high pre-chemotherapy LMR (≥3.11) was an independent favorable prognostic factor for PFS and OS. Additionally, patients whose LMR remained high (high–high subgroup), increased (low–high subgroup), or decreased (high–low subgroup) following chemotherapy showed better results in terms of PFS and OS than patients whose LMR remained low (low–low subgroup) after chemotherapy, suggesting that the change in LMR before and after chemotherapy may predict the benefit of chemotherapy.

Approximately half of all CRC patients develop distant metastasis [3], which poses a huge clinical challenge. In clinical practice, the FOLFOX regimen is the treatment of choice for patients with chemotherapy-naïve mCRC and has favorable toxicity profiles compared with the irinotecan-based combination regimen [4, 5]. However, a significant difference in the response to chemotherapeutic agents has been noted among mCRC patients, suggesting that mCRC is a heterogeneous disease. Hence, considerable strides have been made in seeking prognostic or predictive biomarkers to classify heterogeneous mCRC. Inflammation is profoundly involved in promoting pathogenesis and progression of tumor [6]. Nowacki et al. [18] revealed that a prolonged duration of ulcerative colitis dramatically increased the risk of CRC (*P* < 0.001), whereas the risk could be markedly reduced by anti-inflammatory treatment (*P* < 0.02), suggesting that inflammation may have a profound influence on the pathogenesis of CRC. Lymphocytes play an important role in constraining the proliferation of malignant cells. As a surrogate marker of weak immunity, peripheral blood lymphopenia is associated with poor survival outcomes in patients with nasopharyngeal carcinoma (NPC) [14]. TILs are observed in the tumor microenvironment and reflect an adaptive immune response [6]. The superior survival outcomes associated with high concentrations of TILs in CRC has been well documented [19–21]. Furthermore, growing evidence suggests that CD8^+^ cells and other activated T lymphocytes might suppress metastasis rather than tumor growth [22]. Monocytes can differentiate into macrophages in the tumor microenvironment [6]. Experimental evidence has shown remarkable interactions between tumor cells, macrophages, and blood vessels, facilitating angiogenesis and promoting tumor cell motility, which eventually results in distant metastases [23, 24]. In addition, a survival advantage associated with low levels of peripheral blood monocytes has been observed in NPC patients [14]. Taking the above considerations into account, peripheral blood LMR, which is an indicator of systemic inflammation, becomes an ideal candidate due to the advantage of simplicity, accessibility, and inexpensiveness compared with complex molecular markers.

A large cohort study including 1547 non-metastatic NPC patients showed that higher LMR levels (≥5.22) were significantly associated with longer DFS and OS (*P* < 0.001) [14]. Subsequent multivariate COX proportional hazard analysis confirmed that higher LMR levels remained a significant independent factor for longer DFS (HR = 0.669, 95% CI 0.535–0.838, *P* < 0.001) and OS (HR = 0.558, 95% CI 0.417–0.748, *P* < 0.001) [14]. Lin et al. [16] found that newly diagnosed metastatic non-small cell lung cancer patients with increased LMR (≥4.56) obtained longer PFS (5.60 vs. 5.04 months, *P* = 0.001) and OS (13.20 vs. 11.72 months, *P* < 0.001) than those with decreased LMR. Moreover, LMR was an independent prognostic factor for PFS (HR = 0.660, 95% CI 0.512–0.851, *P* = 0.001) and OS (HR = 0.530, 95% CI 0.409––0.687, *P* < 0.001) [16]. For patients with stage II and III colon cancer, patients with high preoperative LMR (>2.83) showed better results in terms of time-to-recurrence (HR = 0.47, 95% CI 0.29–0.76, *P* = 0.002) and OS (HR = 0.51, 95% CI 0.31–0.83, *P* = 0.007) than those with low preoperative LMR [17]. Similar to these aforementioned reports, our study cohort demonstrated that chemotherapy-naïve mCRC patients with high pre-chemotherapy LMR (≥3.11) showed significant improvements in PFS (9.2 vs. 7.6 months, *P* < 0.001) and OS (19.4 vs. 16.6 months, *P* < 0.001) compared with patients with low pre-chemotherapy LMR. Furthermore, our subsequent COX multivariate analysis showed that high pre-chemotherapy LMR was an independent favorable prognostic factor for PFS (HR = 0.710, 95% CI 0.558–0.903; *P* = 0.005) and OS (HR = 0.662, 95% CI 0.501–0.875, *P* = 0.004). Taking the above considerations into account, pre-treatment LMR seems to be an independent prognostic factor that can classify cancer patients into different prognostic subgroups, improving the personalized management of cancer.

In addition to pre-treatment LMR, the prognostic significance of changes in LMR before and after treatment should also be explored. In our study cohort, the optimal cut-off values of the pre- and post-chemotherapy LMR were both set at 3.11. Subsequently, all patients were divided into four subgroups based on the cut-off values of pre- and post-treatment LMRs. As expected, the low–low subgroup had the worst survival results, whereas the high–high subgroup had the best survival results. Additionally, patients whose LMR increased (low–high subgroup) or decreased (high–low subgroup) after chemotherapy showed longer PFS and OS than patients whose LMR remained low (low–low subgroup) after chemotherapy. A recent study evaluated the prognostic impact of the neutrophil-to-lymphocyte ratio (NLR) in 199 non-smokers with advanced lung adenocarcinoma receiving gefitinib or standard chemotherapy as first-line therapy [25]. In the high pre-treatment NLR group, patients whose NLR decreased after treatment had a longer OS than those whose NLR remained high following treatment (20.7 vs. 7.9 months, *P* < 0.001). Similarly, in the low pre-treatment NLR group, patients whose NLR remained low after treatment had a longer OS than those whose NLR increased after treatment (26.4 vs. 18.9 months, *P* < 0.001) [25]. Therefore, a change in LMR before and after chemotherapy seemed to predict the benefit of chemotherapy in chemotherapy-naïve mCRC patients receiving the FOLFOX regimen.

The strengths of this study are the large sample size and the relatively low heterogeneity of patients who were all diagnosed with chemotherapy-naïve mCRC and treated with FOLFOX chemotherapy. Nevertheless, the major limitation of this study is its retrospective design, which contributes to selection bias. Prospective studies are necessary to validate our results. Additionally, inflammatory factors such as lymphocytes and monocytes may be influenced by many potential confounding factors, such as latent infection and autoimmune disease. Hence, potential confounding factors must be excluded while considering LMR as a prognostic factor for cancer patients.

In conclusion, for patients with previously untreated mCRC receiving FOLFOX chemotherapy, a high pre-chemotherapy LMR is an independent favorable prognostic factor for PFS and OS, and changes in LMR before and after chemotherapy seem to predict the benefit of chemotherapy.
